# Integrated biological and chemical characterisation of a pair of leonardesque canal lock gates

**DOI:** 10.1371/journal.pone.0247478

**Published:** 2021-03-10

**Authors:** Luca Zoia, Anika Salanti, Claudio Giorgione, Rodolfo Gentili, Sandra Citterio, Isabella Gandolfi, Andrea Franzetti, Marco Orlandi

**Affiliations:** 1 Department of Earth and Environmental Sciences, University of Milan-Bicocca, Milan, Italy; 2 Museo Nazionale della Scienza e della Tecnologia “Leonardo da Vinci”, Milano, Italy; University of Trento, ITALY

## Abstract

The Museo Nazionale della Scienza e della Tecnologia “Leonardo da Vinci” in Milan is exposing two pairs of canal lock gates, used to control the water flow in Milan canal system, whose design appears in the Leonardo’s Codex Atlanticus. The wood present in the gates has been deeply characterised by mean of a multidisciplinary investigation involving i) DNA barcoding of wood fragments; ii) microbial community characterisation, and iii) chemical analyses. DNA barcoding revealed that two fragments of the gates belonged to wood species widely used in the middle age: *Fagus sylvatica* and *Picea abies*. The chemical characterisations were based on the use of ionic liquid as dissolving medium in order to analyse the entire cell wall material by means of Gel Permeation Chromatography (GPC) and 2D-NMR-HSQC techniques. This multidisciplinary analytical approach was able to highlight the complex nature of the degradation occurred during the gate operation (XVI-XVIII centuries): an intricate interplay between microbial populations (i.e. *Shewanella*), inorganic factors (i.e. iron from nails), physical factors and the lignocellulosic material.

## Introduction

Recently, a diagnostic campaign funded by “Regione Lombarida” was realized with the goal to achieve useful information on the conservation conditions and historical data of two canal lock gates [1]. Those two pairs of lock gates, whose design appears in the Leonardo da Vinci Codex Atlanticus, were used to control the water flow in Milan canal system during the XVI-XVIII centuries and were removed in XX century from San Marco and Cassina di Pomm locks (Milan). They are nowadays property of the Milan city museums and conserved in the Museo Nazionale della Scienza e della Tecnologia “Leonardo da Vinci”. Together with the radiographic analyses and radiocarbon dating, a preliminary chemical characterisation of wood was performed in order to assess conservation strategies for museum exhibition [1]. Gates were in fact partially waterlogged and subjected to wet/dry cycles producing physical and chemical modifications of wood structure. It is well known that waterlogged woods are artefacts that represent a conservation challenge still far to be solved, due to the complex degradation pathways they undergo [2–8]. A picture as much complete as possible of the chemical composition and degradation of the lignocellulosic materials is the first step in the process of finding innovative and efficient conservation strategies [9–12]. Given the peculiarity of canal lock gates artefacts, existing scientific literature does not report any systematic approach or analytical study on historical canal gates. Moreover, wood is a complex, tridimensional material composed of several polymers interconnected with each other, heterogeneous at macro and micro-scale, and particularly recalcitrant to wet chemical analyses. This is why the wood present in the locks has been deeply characterised by mean of a multidisciplinary investigation involving i) barcoding of wood fragments, ii) microbial community characterisation, and iii) chemical analyses. The barcoding technique is becoming more and more important nowadays because it permits the species’ identification in presence of a limited amount of material and/or in presence of a not integer sample (which usually does not permit the taxonomic identification via microscopic observation): these conditions are common in cultural heritage [13, 14]. Anyways, DNA extraction from archaeological woods is problematic due to the complex nature of the matrix and hydrolytic and oxidative processes that cause DNA degradation. Despite these events, the taxonomic identification and possibly the knowledge about the origin of wood samples are pivotal both studying the chemical mechanisms in action and understanding the history of the artefact [15]. Also the importance of bacterial community, and its interaction with inorganic contaminants and the lignocellulosic material, in archaeological wood degradation and conservation was already recognized [2, 4, 16–21]. DNA barcoding for the identification of wood species and the characterisation of microbial communities could be integrated by the chemical analyses in order to have a complete picture of the history and the state of the artefact. In this paper, the chemical characterisations were based on the use of ionic liquid (IL) as dissolving medium [22, 23] in order to analyse the entire cell wall material in solution state by means of Gel Permeation Chromatography (GPC) and two-dimensional high-resolution nuclear magnetic resonance in heteronuclear single quantum correlation experiment (2D-NMR-HSQC) techniques [9, 12, 14, 23]. Highly substituted lignocellulosic esters were obtained by reacting the solubilised woods with either acetyl chloride or benzoyl chloride [23]. As a result, the functionalised woods developed an enhanced solubility in molecular solvents opening access to typical “wet” techniques such as Gel Permeation Chromatographic (GPC), and 2D-HSQC-NMR spectroscopy [9, 12]. GPC analyses permit the detection of the whole substrate components, which are cellulose, hemicelluloses, and lignin (after benzoylation, [1]) while it is possible to focus on their possible chemical connections (i.e. Lignin Carbohydrates Complexes, LCCs) after acetylation. 2D-HSQC-NMR facilitates a detailed investigation of the chemical structure of archaeological wood samples, by identifying the cross-peak related to polysaccharides (cellulose and hemicelluloses) and to the aliphatic and aromatic domains of lignin. Together with Fourier Transform-Infra Red (FT-IR) spectroscopy, and already performed Scanning Electron Microscopy (SEM), and Maximum Water Content (MWC) measurements [1], this multidisciplinary analytical approach was applied to understand the nature of the degradation occurred during the gate operation (XVI-XVIII centuries).

## Materials and methods

### Materials

**Fig 1:**
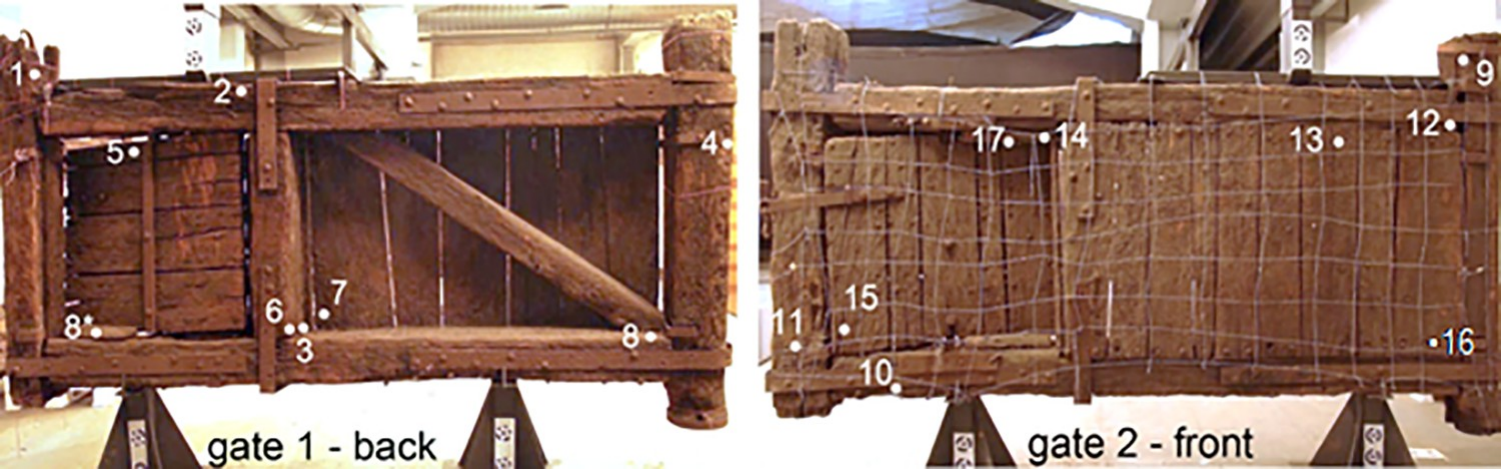
Images of the two canal lock gates. Numbers and points represent the sampling points for the chemical and biological analyses.

All reagents were purchased from Sigma-Aldrich and used as received. Objects of the study were the two canal gates removed in 1935 from San Marco Lock (Fig 1). Until the removal, the gates were waterlogged in the “Naviglio” canal. The gates were removed from the lock in the 1935 under the supervision of Raffaele Cormio, not consolidated, air dried and stored (in controlled but unknown conditions) as part of the Civic Xylotheque (Milan). From 1952 to 2000 they were expose in the “Xylotheque Cormio” in the Museo Nazionale della Scienza e della Tecnologia “Leonardo da Vinci”. Gate 1 has a width of ca. 345 cm and a height of ca. 180 cm, gate 2 has a width of ca. 335 cm and a height of ca. 180 cm. The thickness of the gates varies from 4.5 cm of the planks to 29.0 cm of the vertical bars. Fragments (roughly 15 x 15 x 15 mm) were sampled following the scheme reported in Fig 1, where the white numbers label the sampling points [1]. Depending on the amount of wood, the samples have been submitted to different characterisation described in Table 1.

**Table 1:** Summary of the procedures performed on the San Marco canal lock gates in this work and in [1].

		Samples																	
	Analyses	1	2	3	4	5	6	7	8	8*	9	10	11	12	13	14	15	16	17
**[1]**	**MWC%**	X	X	X	X	X	X	X	X		X			X		X	X	X	
	**SEM**		X	X	X	X	X						X	X	X				
	**GPC Bz**	X	X	X	X	X	X	X	X	X	X	X	X	X	X	X	X	X	X
**This work**	**GPC Ac**	X					X					X	X	X	X				
	**FT-IR**	X					X					X	X	X	X				
	**2D-NMR-HSQC**	X					X					X	X	X	X				
	**BARCODING**		X					X											
	**MICROBIAL COM.**											X				X		X	

### Method

#### Barcoding

The samples 2 and 7 were prepared for barcoding analysis and the subsequent botanical identification. After removing the external parts of the samples with a sterile scalpel (to avoid fungal contamination), some sample slices were washed with distilled water and kept in a sterilized glass bottle with distilled water at 4°C for about 72 h; the water was repeatedly changed at every 12 h. DNA extraction was carried out combining CTAB and DNEasy Plant miniKit (Qiagen, Germany) methods [24]. To 0.50 mg of frozen (in liquid nitrogen) and ground tissue plant material, 900 μl of CTAB extraction buffer (preheated at 65°C), 50 μl of 2-mercaptoethanol and 20 μl of RNAaseA (Qiagen, Germany) were added. Samples were homogenized for 3 min and then incubated for 20 min at 65°C, with gentle shake and finally kept for 8 min at room temperature. The samples with the addition of one volume of chloroform were spinned at 9,000 rpm for 10 min (step repeated until the supernatant became clear). The aqueous phase of samples (upper part) was then placed into a new sterile tube (2 ml) and added with one volume of sterile water. The pH was adjusted to 7.0 with 20% HCl. Subsequently, the lysate filtration, the DNA elution and its suspension in 50 μl of AE buffer were performed by using the QIAshredder and DNeasy spin columns according to the manufacturer’s instructions (Qiagen, Germany). The concentration of DNA was measured using a NanoDrop ND-1000 spectrophotometer (Thermo Scientific, USA). Molecular characterisation was performed with 3 different DNA primer pairs (Pp) widely used in barcoding [25], selected in the rbcL region of the plastid DNA:

- PpA (*rbcL*1–*rbcL*R3A): *rbcL*1 forward (TTGGCAGCATTYCGAGTAACTCC) and *rbcL*R3A reverse (TTCGGTTTAATAGTACAGCCCAAT);
- PpB (*rbcL*F2–*rbcL*R3A): *rbcL*F2 forward (TGTTTACTTCCATTGTGGGTAATG) and *rbcL*R3A reverse (TTCGGTTTAATAGTACAGCCCAAT);
- PpC (*rbcL*1F–*rbcL*724 R): *rbcL*1F forward (ATGTCACCACAAACAGAAAC) and *rbcL*724 reverse (TCGCATGTACCTGCAGTAGC).

Polymerase chain reaction (PCR) amplification was performed using PuReTaq Ready-To-Go PCR beads (Amersham Bioscience, Italy) in a 25 μL reaction according to the manufacturer’s instructions. PCR cycles consisted of an initial denaturation step for 7 min at 94°C, 35 cycles of denaturation (45 s at 94°C), annealing (30 s at 48°C) and elongation (1 min at 72°C), and a final extension at 72°C for 10 min. PCR products were sequenced using an ABI 3730XL automated sequencer at BMR Genomics (Padua, Italy). Manual editing of raw traces and subsequent alignments of forward and reverse sequences allowed us to assign edited sequences to species. Particularly, the 3′ and 5′ terminals were clipped to generate consensus sequences for each taxon. In order to avoid the inclusion of inadvertently amplified nuclear pseudogenes of plastid origin (see, for example, De Mattia et al. [26]), barcode sequences were checked following the guidelines of Buhay, 2009 [27]. The *rbcL* sequences were visualised and edited using the Sequencer 4.8 program, and a sequence similarity search, for plant identification was carried out by querying the GenBank database (GenBank accession numbers: MT231324-MT231327), using BLAST program (https://blast.ncbi.nlm.nih.gov/Blast.cgi) [28]. The sequences obtained were then deposited in the NCBI’s data library (https://www.ncbi.nlm.nih.gov/; submission #2323851).

#### Microbial community characterisation

Microbial communities hosted by three wood samples (samples 11, 14 and 16) were characterised by high-throughput sequencing of the taxonomic markers 16S rRNA gene for bacteria and ITS1 for fungi. Total genomic DNA was extracted from approximately 150 mg of wood for each sample, using the FastDNA™ SPIN Kit for Soil (MP Biomedicals, Solon, OH, USA). Extraction was performed according to manufacturer’s instructions, except that the FastPrep® instrument was run for 45 s at a speed of 6.5, and the following centrifugation step was extended to 15 min. The V5-V6 hypervariable regions of 16S rRNA gene were PCR-amplified using 783F and 1046R primers [29, 30], while ITS1 region was amplified with ITS1F and ITS2 primers [31]. At the 5’ end of each primer, a 6-bp barcode was included to allow sample pooling and sequence sorting. All amplicons were sequenced by MiSeq Illumina (Illumina, Inc., San Diego, CA, USA) with a 2 × 250 bp paired-end protocol. For each sample, 3 × 75 μL volume PCR reactions were performed with Phusion® High-Fidelity DNA Polymerase (New England Biolabs, Ipswich, MA, USA), using 5X Phusion GC Buffer, MgCl_2_ at a final concentration of 2 mM, 200 μM of each dNTP, 0.5 μM of each primer, and 1.5 U of Phusion polymerase. The cycling conditions for the amplification of the 16S rRNA gene fragment were: initial denaturation at 98°C for 1 min; 28 cycles at 98°C for 7 s, 47°C for 20 s, and 72°C for 10 s, and a final extension at 72°C for 5 min. The cycling conditions for the amplification of the ITS1 region were: initial denaturation at 98°C for 1 min; 30 cycles at 98°C for 7 s, 50°C for 20 s, and 72°C for 10 s, and a final extension at 72°C for 5 min. However, no PCR products were obtained in reactions using ITS primers; therefore, further analyses were conducted on bacterial communities only. The amplicons were purified with the Wizard® SV Gel and PCR Clean-up System (Promega Corporation, Madison, WI, USA) and purified DNA was quantified using Qubit® (Life Technologies, Carlsbad, CA, USA). Further library preparation with the addition of standard Nextera indexes (Illumina, Inc., San Diego, CA, USA) and sequencing were carried out at Parco Tecnologico Padano (Lodi, Italy). Reads from sequencing were demultiplexed according to the indices. Uparse pipeline was used for the subsequent elaborations [32]. Forward and reverse reads were merged with perfect overlapping and quality filtered with default parameters. Suspected chimeras and singleton sequences (i.e. sequences appearing only once in the whole data set) were removed. OTUs were defined on the whole data set clustering the sequences at a 97% of similarity and defining a representative sequence for each cluster. The abundance of each OTU was estimated by mapping the sequences of each sample against the representative sequence of each OTU at 97% of similarity. Taxonomic classification of the OTU representative sequences was obtained by RDP classifier. Sequences classified as chloroplasts were discarded.

#### FT-IR

Chemical composition of the archaeological wood powders (Table 1) was investigated by means of a Fourier Transform Infrared (FT-IR) spectrometer (Nicolet iS10, Thermo Scientific) equipped with an ATR sampling accessory with a diamond crystal (Smart iTR). For each spectrum 64 scans, with a spectral resolution of 4 cm^-1^, were recorded.

#### Wood acetylation in ionic liquid

Acetylation reaction was performed in 1-allyl-3-methylimidazolium chloride ([amim]Cl, 950 mg), on the wood powders (70 mg, Table 1) with acetyl chloride, as reported by Salanti [23]. The procedure was slightly modified and at the end of the reaction, 200 μL of iodomethane were added and left to react for 15 minutes extra in order to convert the carboxylic acids into methyl esters. The acetylated wood samples were solubilized in THF (1 mg mL^-1^) for GPC analysis and in d_6_-DMSO for NMR analyses (50 mg mL^-1^).

#### GPC analyses

Acetylated wood samples after dissolution in THF (1 mg mL^-1^) were analysed by GPC using THF as eluent at a flow rate of 1 mL min^-1^. The analyses were performed on an HP1100 liquid chromatography system equipped with an UV-Vis detector set at 280 nm. The injection port was a Rheodyne® equipped with a 20 μL loop. The GP-column system was composed as follows (according to the solvent flow direction): Agilent PLgel 5 μm, 500 Å, Agilent PLgel 5 μm, 1000 Å, and Agilent PLgel 5 μm, 10000 Å. PL Polymer Standards of Polystyrene from Polymer Laboratories were used for calibration. The peak molecular weight (M_p_) values reported are the average of three replicate analyses (M_p_: ±100 g mol^-1^, P = 0.05, n = 3).

#### 2D-HSQC-NMR analyses

Two-dimensional Heteronuclear Single Quantum Coherence spectra (2D-HSQC) were run in DMSO-d_6_ on IL-acetylated wood samples. The inverse detected ^1^H-^13^C correlation spectra were measured on a Bruker Avance 500 MHz spectrometer set at 308 K. The spectral width was set at 5 kHz in F2 and 25 kHz in F1. In total 128 transients in 256 time increments were collected. The polarization transfer delay was set at the assumed coupling of 140 Hz, and a relaxation delay of 2 s was used. The spectra were processed using Π/2 shifted squared sinebell functions in both dimensions before FT.

**Fig 2:**
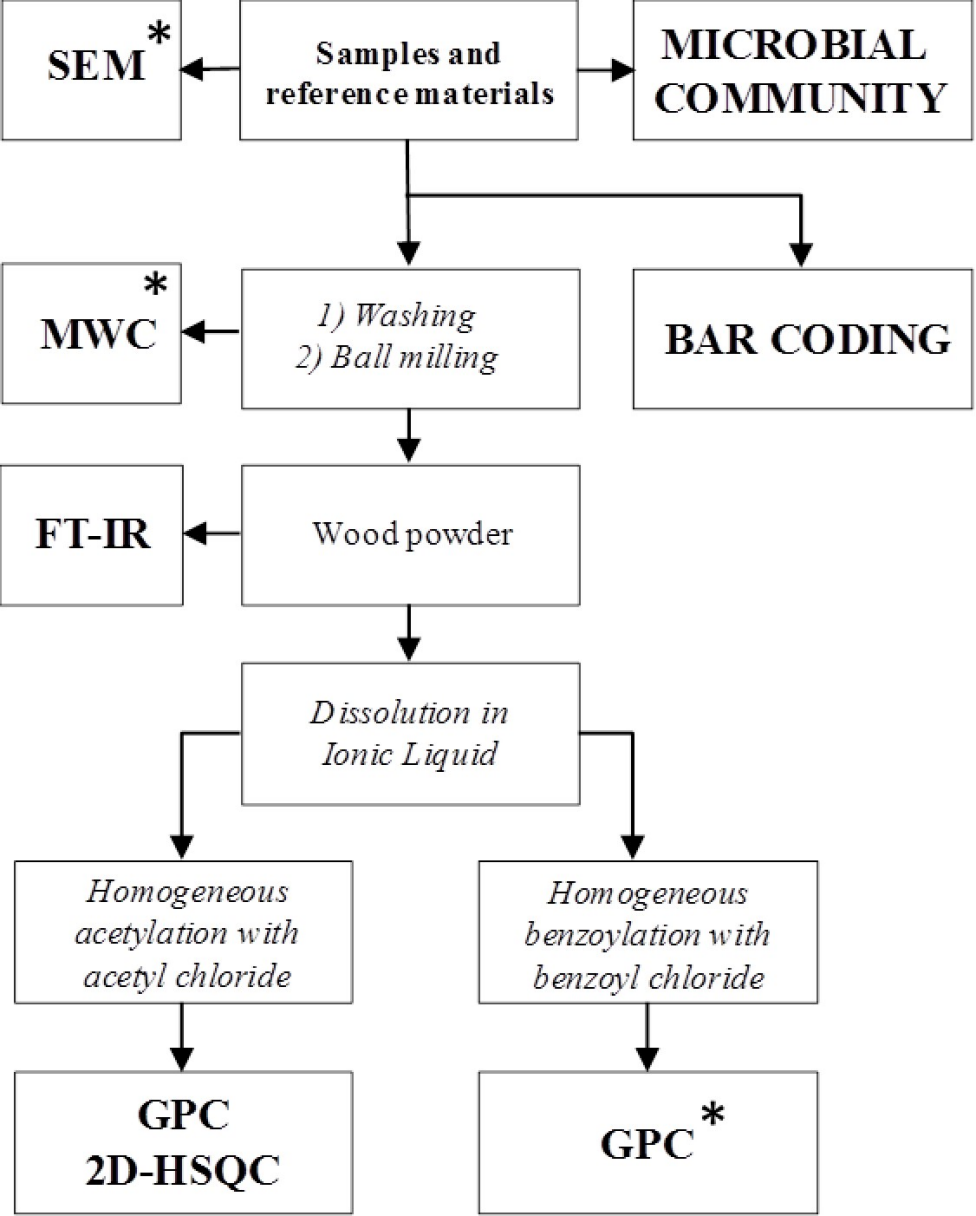
The integrated characterisation approach performed in this work and in [1]*.

The integrated procedures are schematised in Fig 2 along with the SEM, MWC and GPC after benzoylation analyses performed in [1].

## Results

### Plant DNA barcoding

DNA extraction from ancient wood fragments was successful for the two investigated samples (2 and 7) with good DNA quality but modest yield (i.e. 5–10 ng mL^-1^). Differences in amplification success, PCR product lengths and sequence quality were detected for the three considered *rbcL* loci (PpA, PpB and PpC). In particular: a) the PpA products ranged from 361 to 376 bp, for the samples 2 and 7, respectively; b) the PpB products ranged from 128 to 136 bp, for the samples 7 and 2, respectively; c) the PpC products failed to yield usable sequences. The BLAST results based on sequence matching as well as the putative species identification produced the following results:

- Sample 2, PpA: matched with *Fagus sylvatica* L. with a percent identity of 99.72;
- Sample 2, PpB: matched with *Fagus sylvatica* L. with a percent identity of 99.52;
- Sample 7, PpA: matched with several species of the genus *Picea* with a percent identity of 99.20 (including the European species *Picea abies* (L.) H. Karst.);
- Sample 7, PpB: matched with several species of the genus *Picea* with a percent identity of 98.45 (including the European species *Picea abies* L.).

### Microbial community

**Fig 3:**
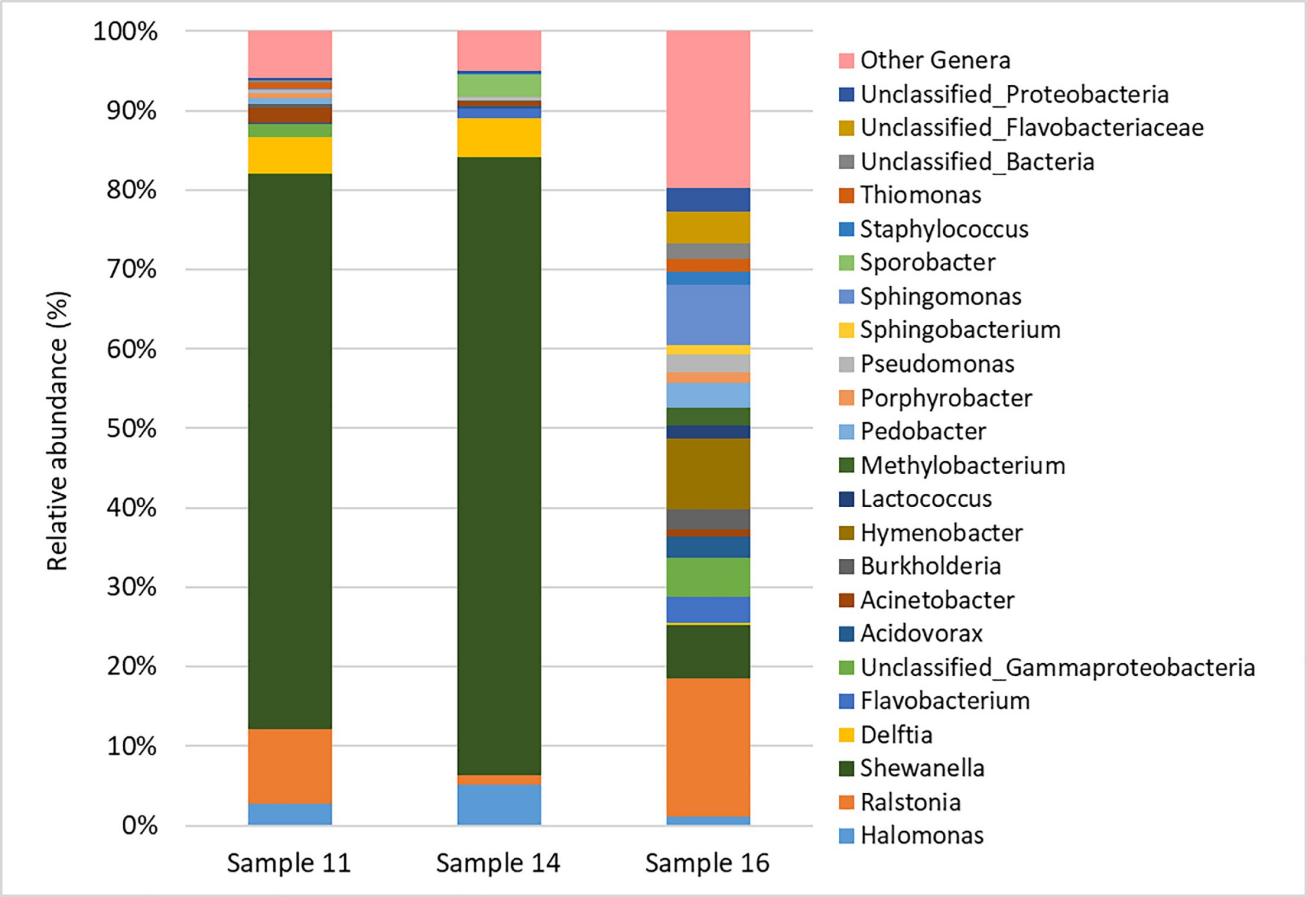
Taxonomic classification of bacterial communities associated to wood samples 11, 14 and 16 at Genus level. “Other Genera” groups genera that were less abundant than 1% in all samples.

Bacterial communities of samples 11 and 14 were clearly dominated by *Shewanella* (70.0 and 77.8%, respectively, Fig 3). This genus was also present in sample 16, although at a much lower abundance (6.8%). *Delftia* and *Halomonas* were also more abundant in samples 11 and 14 than in sample 16. In fact, *Delftia* had a relative abundance of 4.6 and 4.9% in samples 11 and 14, respectively, but only of 0.3% in sample 16, while *Halomonas* had a relative abundance of 2.8, 5.1 and 1.1% in the three samples, respectively. In contrast, bacterial community of sample 16 was more diverse and was not clearly dominated by any populations. Here, the most abundant genera were *Ralstonia*, *Hymenobacter*, and *Sphingomonas*, with relative abundances of 17.4, 8.9 and 7.5%, respectively. However, several other genera were part of bacterial community of this sample, as well as unclassified members of the class Gammaproteobacteria and of the family Flavobacteriaceae. By contrast, no amplicons were obtained in PCR reactions using ITS primers. This was possibly due to the very low amount of total DNA extracted from the wood samples, of which fungal DNA is only a fraction. Therefore, although the presence of fungal communities in gate wood cannot be excluded, it was not possible to fully characterise them.

### Chemical characterisation

**Fig 4:**
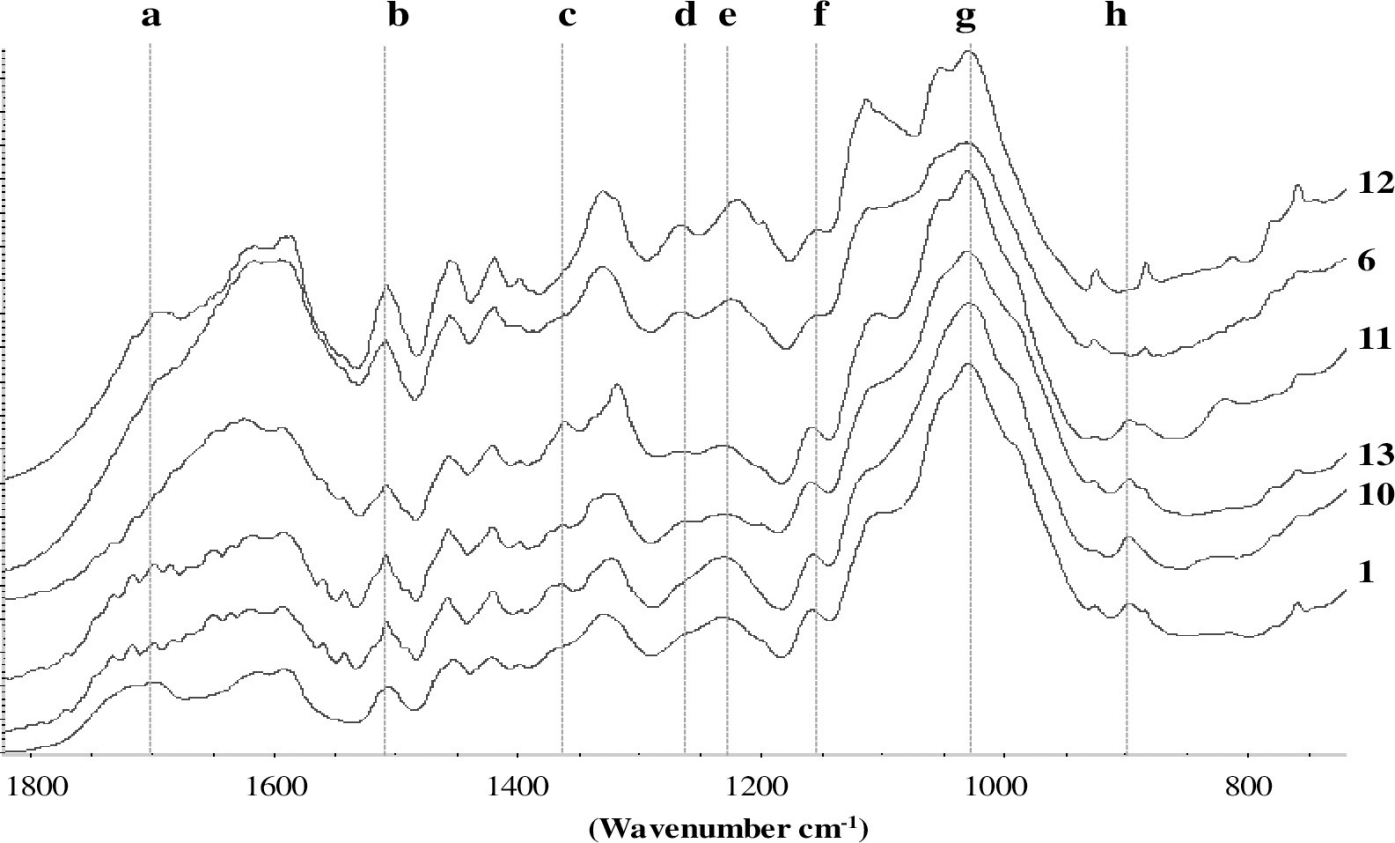
FT-IR spectra (range 1800–700 cm^-1^) for wood samples 1, 10, 13, 11, 6 and 12 along with the main band assignments (in letter) reported in Table 2.

As a preliminary characterisation, the wood powders have been submitted to FT-IR analyses. The stacked FT-IR spectra of the samples 1, 10, 13, 11, 6 and 12 were reported in Fig 4 while in Table 2 the assignments of the main lignocellulosic bands are described.

**Table 2:** Assignments of the FT-IR bands reported in Fig 4.

Band	Wavenumber (cm^-1^)	Assignment
**a**	1730	Unconjugated C = O in xylans (hemicellulose)
**b**	1505	Aromatic skeletal in lignin
**c**	1375	C–H deformation in cellulose and hemicellulose
**d**	1270	Guaiacyl ring breathing lignin
**e**	1240	Syringyl ring and C–O stretch in lignin and xylan
**f**	1157	C–O–C vibration in cellulose and hemicellulose
**g**	1030	C–O stretch in cellulose and hemicellulose
**h**	900	C–H deformation in cellulose

**Fig 5:**
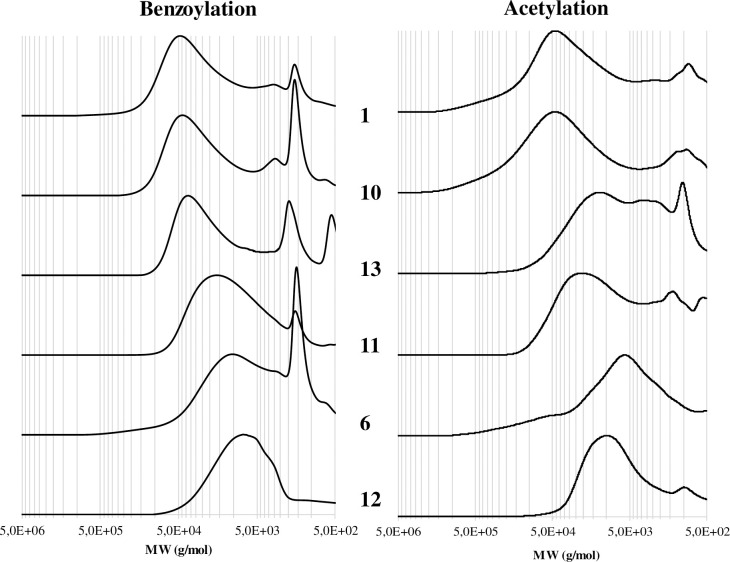
Gel Permeation Chromatography (GPC-UV) profiles (benzoylated on the left at 240 nm, acetylated on the right at 280 nm) for the 1, 10, 13, 11, 6 and 12 woods samples. Y-scale in A.U. normalized.

The spectra for the sample 1, 10, 13 were qualitatively similar, where it was possible to detect the principal bands related to lignin, hemicelluloses and cellulose [17, 18]. The spectra of the sample 6 and 12 were instead different, with the lignin bands at 1505 (**b**) and 1270 cm^-1^ (**d**) enhanced in relation to the reduction of the typical bands of polysaccharides at 1375 (**c**), 1157 (**f**), 1030 (**g**) and 900 (**h**) cm^-1^. However, different lignocellulosic components have overlapped absorption bands: for example, the band (**a**) at 1730 cm^-1^ related to the C = O unconjugated in xylans, was present in the sample 1, 10 and 13. This band was missing in the sample 11 and then increased again in the sample 6 and 12, probably due to the oxidation occurred to lignin with the formation of carbonyl groups. In addition, also the band (**d**) at 1240 cm^-1^ (syringyl ring and C–O stretch in lignin and xylan) had a non-linear trend decreasing from the sample 1 to 11 and then increasing again in the sample 6 and 12. In general, the FT-IR spectra highlighted a degradation typical of waterlogged woods consisting in the relative enrichment on lignin due to the loss of the polysaccharides [10, 11]. Then we adopted ionic liquids (ILs) as non-derivatizing solvents allowing us to overcome the difficulty of dissolving wood in conventional molecular solvents [22]. Benzoylated (performed in [1]) and acetylated wood samples were analysed by GPC at 240 and 280 nm, respectively, in order to maximize their analytical response. The chromatograms obtained are reported in Fig 5.

**Fig 6:**
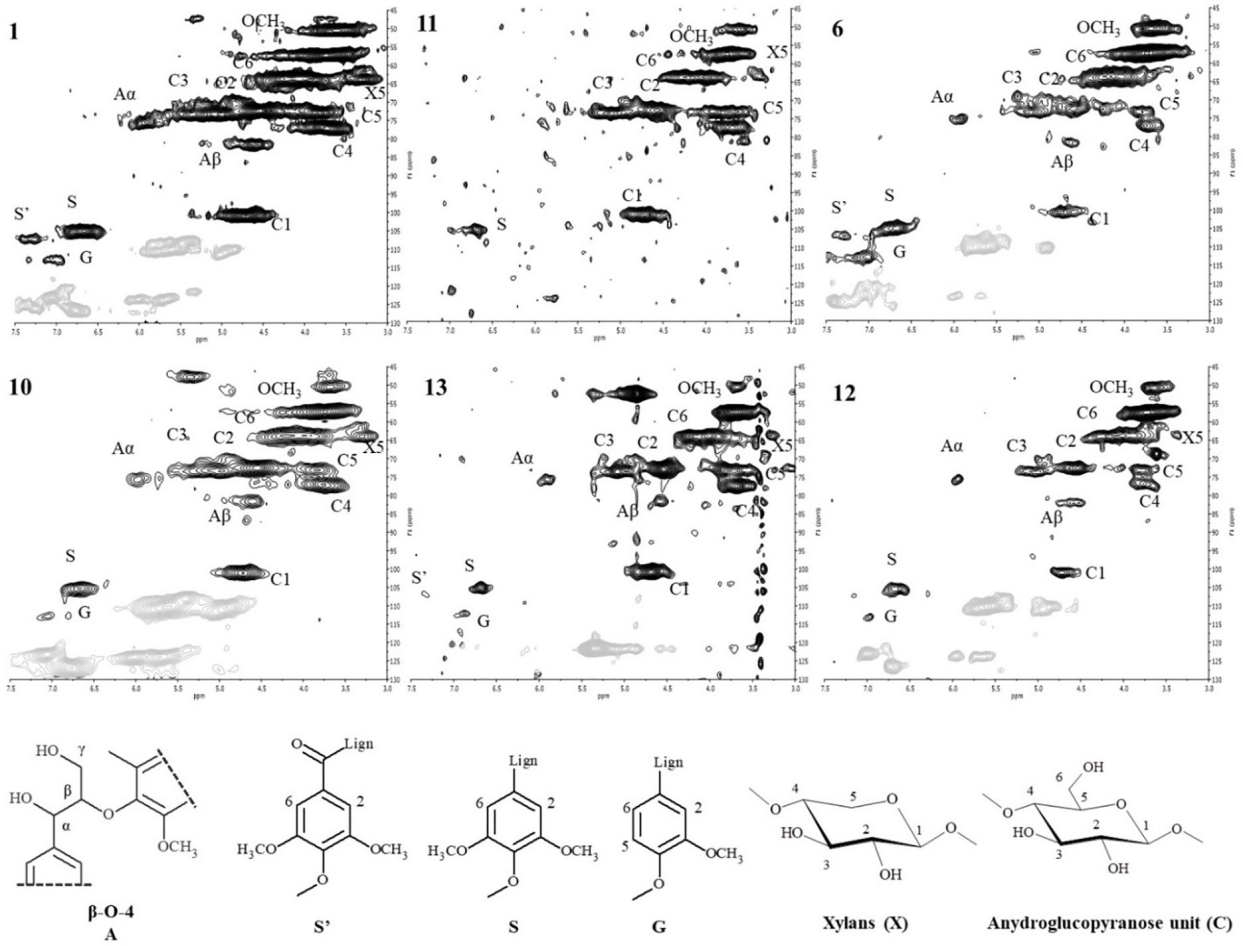
2D-HSQC-NMR spectra of acetylated wood samples (1, 10, 11, 13, 6, 12). At the bottom, the main aliphatic and aromatic lignin and polysaccharide structures are reported: A) β-O-4 alkyl-aryl ethers; S’) oxidized syringyl units bearing a carbonyl at Cα; S) syringyl units; G) guaiacyl units; OCH_3_ methoxy group; X_n_) xylan units of hemicelluloses; C_n_) anydroglucopyranose units of cellulose.

As previously reported [23], after benzoylation reaction in IL, polysaccharides and lignin have a similar UV response (240 nm) due to the presence of the phenyl ester. Therefore, the chromatograms of benzoylated samples reported the molecular weight distribution of the whole cell wall components. On the other hand, the chromatograms of acetylated samples (280 nm) account exclusively for the molecular weight distribution of those lignocellulosic fractions that naturally contain aromatic moieties (mainly LCCs and lignin). In this view, it is possible to observe a trend in degradation related to the decreasing of the molecular weights from sample 1 to sample 12 (Fig 5, left panel). In particular, samples 1, 10 and 13 had a similar molecular weight distribution in line with sound hardwood (not reported) [12]. On the contrary, samples 11, 6 and 12 had a GPC profile shifted to lower molecular weights: this was probably due to the cellulose hydrolysis. It is possible to observe a similar and general trend for the GPC profiles after acetylation (Fig 5, right panel). However, some differences could be highlighted: samples 1 and 10 had a GPC acetylated profile typical of undegraded woods, while the molecular weight distribution of the sample 13 showed degradation on the LCCs structure. Sample 6 and 12 were characterised (as for benzoylation) by a GPC profile shifted to low molecular weights. The sample 11 had behaviour not in line with the general trend. This trend, already reported for waterlogged woods, could be rationalised in different temporal phases: i) LCCs degradation with partial loss of hemicellulose, ii) unshielded cellulose degradation with relative enrichment in lignin [9]. The high solubility achieved after wood acetylation enables the analysis of derivatized wood by means of 2D NMR techniques after dissolution in DMSO-d_6_ [12]. The HSQC spectra of samples 1, 10, 11, 13, 6 and 12 along with the main chemical structures for lignin, hemicelluloses and cellulose are reported in Fig 6. Considering the results from the barcoding analyses, it is possible to hypothesize that all the samples have the same botanical origin, *Fagus sylvatica*: in fact in all the samples, the hardwood diagnostic peak of the syringyl unit was detected. As already observed by FT-IR and GPC, the samples 1 and 10 were characterised by a HSQC spectrum typical of a well-preserved wood. From a chemical point of view, the main components were detected: cellulose (indicated as C_n_ for the anhydroglucopyranes unit), hemicelluloses (indicated as X_5_, considered representative for glucuronoxylans) and lignin (indicated as A for aliphatic and S, S’ and G for aromatic region). The NMR data were also in agreement with FT-IR and GPC for the samples 13, 6 and 12: those samples were characterised by a weak X_5_ signal (samples 13 and 12) or even not detected (sample 6), and the cellulose C_n_ signals were in general less intense. The results indicated a trend in lignin enrichment via LCCs degradation. The sample 11 was on the contrary characterised by the presence of the X_5_ signal and the disappearing of the lignin signals A (aliphatic region).

## Discussion

**Fig 7:**
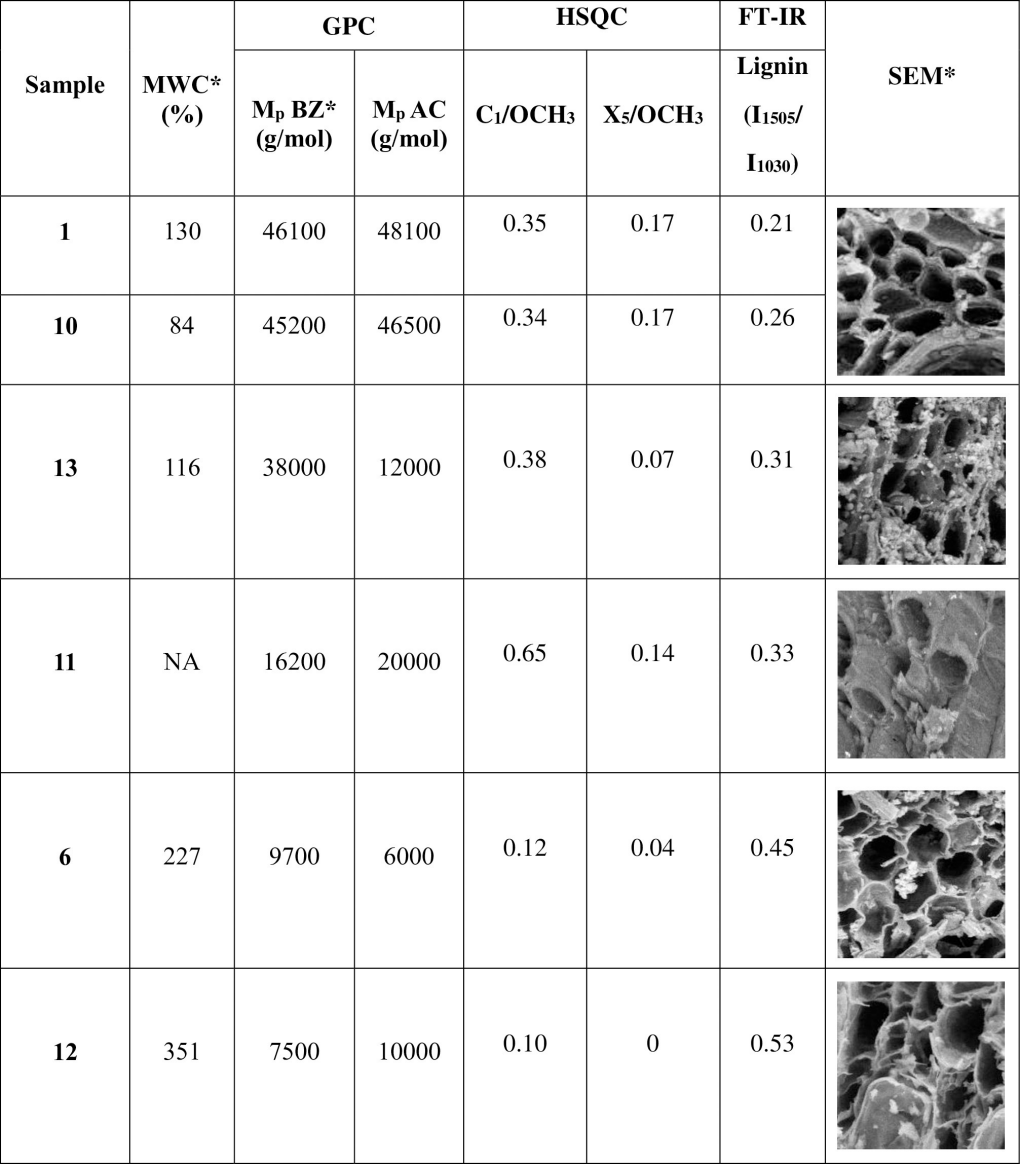
MWC (%), GPC data output (M_p_ in mol/g for benzoylated BZ and acetylated AC profiles), integration ratio C_1_/OCH_3_ and X_5_/OCH_3_ from HSQC spectra, intensity ratio 1505/1030 cm^-1^ from FT-IR spectra, and SEM images for the archaeological samples 1, 10, 13, 11, 6 and 12. * Analysed in [1].

Remarkably, our results showed that samples were 2 and 7 were attributed, by means of barcoding tools, to two very common species at the regional level, in the Lombardy pre-Alps and Alps that can be supposed as the origin areas of the woods: a) *F*. *sylvatica* (beech) ranges from about 500 to 1200 m a.sl.; b) *P*. *abies* (Norway spruce) ranges from about 800 to 2200 m a.s.l.. Since roman times and through the Middle Age, the woods of these two species have always been economically important both as structural woods and for their use in domestic and industrial products [33–35]. These considerations were consistent with the radiocarbon dating of the lock, around the XVI-XVIII centuries and with SEM analyses where the image of samples 2 highlighted the typical vessel structures of hardwood [1]. The wood of beech is heavy, hard, highly resistant to shock and suitable for steam bending [36]. Those characteristics explain why this wood species was probably selected to constitute the frame of the canal lock gates (sample 2, Fig 1). On the other hand, the wood of Norway spruce is moderately lightweight, strong, stiff, tough, and hard [37, 38]. Since it can be easily processed and used, the spruce wood was probably selected to constitute the central plank and the shutter (sample 7, Fig 1): those parts could have been often subjected to maintenance during the canal lock gate working. As already reported in [1], the MWC, SEM and GPC after benzoylation analyses of the samples 1–17 indicated a good state of preservation of the wood composition and a deterioration typical of waterlogged woods (mainly on the external part) that depends on the continual filling and emptying of the lock. Radiographic analyses evidenced some metal inclusions probably due to the presence of the metal nails. In waterlogged conditions, the oxidation of nails could have generated cations able to catalyse the wood degradation. Depending on the available amount of wood, the samples 1, 10, 13, 11, 6 and 12 have been submitted to different and deeper characterisations following the scheme reported in Fig 2 and Table 1. The main results from the chemical characterisation were resumed in Fig 7 where MWC (%) [1], GPC data output (M_p_ in mol/g for benzoylated and acetylated profiles), integration ratio C_1_/OCH_3_ and X_5_/OCH_3_ from 2D-HSQC spectra, intensity ratio 1505/1030 cm^-1^ from FT-IR spectra [1, 9, 17, 18], and SEM images [1] for the archaeological samples 1, 10, 13, 11, 6 and 12 were reported. The integration ratio C_1_/OCH_3_ and X_5_/OCH_3_ from HSQC spectra were used to quantify respectively the holocellulose/lignin and hemicellulose/lignin ratios. For what that concern MWC values, we need to highlight that the samples were recovered from the surface of the gates which were stored for a long time in dry condition: they could have suffered of collapses that inhibit a complete re-hydration, so the MWC reported were used as indicative values. It is possible to observe that from sample 1 to sample 12 (excluding the sample 11) there was a trend. Samples 1 and 10 were characterized by low MWC, high M_p_ (either benzoylated and acetylated), high C_1_/OCH_3_ ratio, low I1505/I1030 ratio and high X_5_/OCH_3_ ratio. SEM images highlight a thick cell wall. Those results are in agreement with a well-preserved wood chemical structure and composition. Porosity was in the range of sound woods, the cellulose molecular weights indicated absence of hydrolysis, LCCs were intact and no enrichment in lignin was detected. On the contrary, samples 6 and 12 were characterized by MWC indicating low-medium degradation, low M_p_ (either benzoylated and acetylated), low C_1_/OCH_3_ ratio, high I1505/I1030 ratio and low X_5_/OCH_3_ ratio. SEM images highlighted thin cell wall. Those results indicated wood degradation for what that concerns the chemical structure and the lignocellulosic composition. Cellulose was hydrolysed and the small fragments lixiviated, LCCs were degraded with loss of hemicelluloses: the final material resulted on the formation of a spongy lignocellulosic material enriched in lignin content.

The sample 13 was characterized by output data in between samples 1, 10 and 6, 12. It was characterized by low MWC, high M_p_ after benzoylated but low M_p_ after acetylated, high C_1_/OCH_3_ ratio, low I1505/I1030 ratio and low X_5_/OCH_3_ ratio. SEM images highlight a thick cell wall. From a chemical point of view, the sample 13 showed a well-preserved structure but an incoming degradation on LCCs that could be considered the first step for further modifications to cellulose [9]. This degradation could be caused by different physical and chemical factors: i) the conditions at which the artefact was subjected during the working, in particular the dry/wet cycles [1], ii) the role of iron oxidation from nails [1, 39, 40]. Through the microbial ecology analyses, the biological factors were also evaluated. No fungal populations were detected in microbiological characterisation and many bacterial genera found were ubiquitous. Sample 16 hosted a bacterial community very different from those of samples 14 and 11. Particularly, the latter were characterised by a low biodiversity and a strong dominance by one bacterial genus only, *Shewanella*. It can be hypothesized that wood surface of these samples offered harsher conditions to bacterial colonization, thus selecting for more specialised bacteria. In contrast, wood of sample 16 did not seem to exert strong selective pressures on bacterial populations. In fact, many bacterial genera of this community are either ubiquitous in the environment, such as *Ralstonia*, *Flavobacterium*, *Pedobacter*, *Acidovorax* and *Pseudomonas* [41], or are commonly retrieved as associated to aerial parts of many plant species, such as *Hymenobacter*, *Sphingomonas*, *Methylobacterium* and *Burkholderia* [42, 43]. This peculiar bacterial community structure is in agreement with the particular chemical characterisation data, at least for sample 11. In fact, the sample 11, unlike all the other samples, was characterized by the M_p_ acetylated higher than M_p_ benzoylated, high C_1_/OCH_3_ ratio, medium-low I1505/I1030 ratio and high X_5_/OCH_3_ ratio. Those data were not in line with the general trend observed for all the other samples, which were characterised by the enrichment on lignin as degradation marker. The chemical characterisation indicated a partial degradation of polysaccharides (cellulose) but the sample 11 seemed predominantly characterised by the loss of lignin. In fact we also detected the disappearing of the lignin signals A of the aliphatic region, related to the β-O-4 inter-monomeric linkage. Members of the genus *Shewanella*, which was strongly dominant in bacterial community hosted by sample 11, are facultative anaerobic bacteria widely distributed in marine and freshwater environments [44]. They have been widely described as electroactive microorganisms, being able of extracellular electron transfer [45]. Particularly, they can reduce Fe(III) by transferring electrons through soluble shuttles that are either secreted by the cell, such as flavins, riboflavins [46] and melanin [47] or found in the extracellular environment, such as humic acids [48]. The effectiveness of humic acids in this process has been attributed to their high content in polycondensed and conjugated aromatic moieties, which mediate Fe(III) reduction [49]. Analogously, it has been shown that lignin possesses redox activity and can be repeatedly switched between oxidized and reduced states [50]. It can be therefore hypothesized that *Shewanella*, in the presence of Fe(III), may exploit lignin as an electron shuttle, thus finding a suitable environment in wood surface and gaining a selective advantage over other bacteria. In the operation conditions, the repeatedly switching between oxidized and reduced states could lead to a dissimilatory lignin depletion [51]. In contrast, both *Delftia* and *Halomonas* have been described as potential lignin degraders. In fact, it has been proposed that some *Delftia* strains might be able to mineralize lignin-derived aromatic compounds [52], while *Halomonas meridiana* M11 was a part of a lignocellulose-degrading consortium [53]. Moreover, the presence of *Halomonas*, a halophilic and halotolerant microorganism, suggests that locks came in contact with saltwater in their past, at least occasionally. The hypothesis of lignin degradation in archaeological waterlogged wood by a particular bacterial genus such as *Shewanella* must be more deeply investigated.

## Conclusion

In conclusion, the multidisciplinary analytical approach, based on barcoding of wood fragments, microbial community study and chemical analyses, was able to highlight the complex history of the canal lock gates (XVI-XVIII centuries). The DNA barcoding used as innovative technique permitted the identification of the botanical origin revealing that two fragments of the gates belonged to wood species widely used in the middle age: *Fagus sylvatica* and *Picea abies*. The microbial ecology was also investigated identifying the bacterial communities that colonized the gates during their utilization. Both those data were integrated by the chemical characterisation in order to have a complete picture of the history and the state of the artefact: a typical waterlogged wood degradation was confirmed. Anyways for one sample, an interesting different degradation pathway was supposed: an intricate interplay between a particular microbial community with a strong dominance by one bacterial genus only (*Shewanella*) and the presence of iron cation from the oxidation of nails that lead to the lignin depletion in wood.
